# Chemistry, Real and Examinational

**Published:** 1897-02-13

**Authors:** 


### The Hospital

A Journal of

The Medical Sciences and Hospital Administration.

Vol. XXI.—No. 542.] [Feb. 13, 1897.

### CHEMISTRY, REAL AND EXAMINATIONAL.

The Technical Education Board of the London County Council has just concluded a piece of good work, and has moved forward in a direction in which medical and other scientific educators will perforce be obliged to follow. A Committee of the Board, consisting of Dr. Russell, F.R.S. (Chairman), Dr. Ludwig Mond, F.R.S., Professor Ramsay, F.R.S., Mr. C. M. Stuart, Head Master of St. Dunstan's College, Catford, Sir Philip Magnus, and Professor James Stuart, has framed a report embodying certain novel and most significant recommendations on the teaching of chemistry, and incidentally on chemistry in its relation to the arts, and on scientific and technical chemistry as a whole. The report, signed by the Chairman and Secretary of the Technical Education Board, has just been issued. Concerning the report the *Times* says: "Of all the work hitherto done by the Board probably none is of more fundamental importance, as it will give help in a direction in which help has long been sorely needed, and of which there was little prospect or hope; and, coming from such a source, the report is a peculiarly valuable contribution to the inquiry which is now being actively prosecuted into methods of education with the object of ascertaining which are the most likely to promote technical progress."

Technical education lies at the very root, not only of progress, greatness, happiness, and the like, but even of existence itself. Let us then speak and think with all the seriousness and sense of which we are capable of "technical education."

Chemistry is a technical subject; it is likewise a subject of great importance in the arts and in the practice of medicine. What does this important committee, what do the individual members of it, say of the teaching of chemistry—of its teaching as they have been accustomed to see it in this country? Let those who think school teaching should all be technical, and let those also who think it should be mainly educational and non-technical, weigh well what these competent and highly technical persons have thought, and still think, on the subject. Dr. Ludwig Mond, F.R.S., expressed the opinion that "the teaching of technological chemistry in schools or similar institutions was of no value whatever, either for chemical industries, or for the pupils." He advised the London School Board to "confine its energies to the teaching of scientific chemistry as an educational discipline." Did, then, these men of science hold that there is no value in, no necessity for, teaching so difficult a science and art as chemistry technically? They would have been most foolish, most unpractical, most untrustworthy, if they had. Their points are these: Teach chemistry, in the first instance, as an educational subject; as a means of mental training in observation, in accuracy, in judgment, in the art of dealing with tangible and visible things. Teach that to the average boy, to all and sundry. But some of the undifferentiated among the pupils will in due course differentiate themselves as boys with a taste for, with aptitude in, chemistry. Let these be selected out; let them be pushed forward into special schools of chemistry, into colleges and workshops, and finally into manufactories and other like establishments, where real chemical knowledge and real chemical aptitudes will lead to real definite results in the advancement of knowledge, in the improvement of manufactures, in new discoveries, and in the progress and happiness of the race.

Those are the objects at which teaching should aim in chemistry; and not in chemistry only, but in all the sciences, both in Board Schools and everywhere else where science may be taught. But there are methods of teaching as well as objects; and methods can ruin everything if they be stupid or ineffectual. We are most glad to see that this practical and highly scientific committee has put its finger firmly down upon one weak spot in our modern system of education. Examinations, this committee holds, are the very *anathema* of our modern schools and schemes of education. On the question of selecting from among ordinary pupils boys to whom it will be worth while to give a further training in chemistry, what do they say? They say, "We are strongly of opinion that such a selection cannot be satisfactorily made by any ordinary system of examination, but could be made by the head master or some other officer of the school who has personal knowledge . . . of the scholar's school career. . . . We are much impressed by the evils arising from the too frequent examination of young students." Examinations, they advise, should, throughout the whole of the educational life of the pupil, be reduced to the lowest possible minimum.

In a word, the points are these: [Education is one thing; technical training another. Let a boy first be educated, then he will bring an educated intelligence to bear upon technical work. When he comes to technical work, let the idea of mere education be forgotten; let him learn his work like a workman. Whether the subject be chemistry, or biology, or any other science, when it is used as an instrument of education, let all idea of technology be forgotten; when the time arrives for business as distinguished from education, then let the whole soul be given to that which is technical, and let the idea of education be forgotten. In other words, a single purpose, carried out at a single time, will be more profitable in the long run for many purposes than the mixing up of many purposes, and the probable ineffectual carrying out of any.

